# Understanding consumer response to front-of-package labeling: insights from a nationwide survey in Sri Lanka

**DOI:** 10.1186/s12889-025-24994-1

**Published:** 2025-10-31

**Authors:** Millawage Supun Dilara Wijesinghe, Nissanka Achchi Kankanamalage Ayoma Iroshanee Nissanka, Upeksha Gayani Karawita, Balangoda Muhamdiramlage Indika Gunawardana, Weerasinghe Mudiyanselage Prasad Chathuranga Weerasinghe, Vithanage Chandima Nayani Vithana, Kanchana Lanka Kumari Mahagamage, A.M.A.A.P Alagiyawanna, Ranjith Batuwanthudawe

**Affiliations:** Health Promotion Bureau, No.02, Kynsey Road, Colombo 10, Colombo, Sri Lanka

**Keywords:** Noncommunicable diseases, Food labeling, Consumer behavior

## Abstract

**Background:**

Non-communicable diseases are the leading cause of global mortality, with unhealthy diets being a significant risk factor. Front-of-package labelling, particularly the traffic light labeling system, has been proposed as a public health intervention to promote healthier dietary choices. This study investigated consumer awareness, understanding, and responses to the traffic light labeling system in Sri Lanka, where non-communicable diseases account for 83% of deaths.

**Methods:**

This descriptive cross-sectional study was conducted between November 2022 and April 2023 and involved 2,569 participants from 25 districts in Sri Lanka. Multistage cluster sampling was performed to ensure the representativeness of the sample. Data were collected via a self-administered online questionnaire in Sinhala, English, and Tamil, which assessed sociodemographic factors, knowledge, attitudes, and purchasing behaviours related to the traffic light labeling system. Ordinal regression analysis was used to identify the factors influencing adherence to traffic-light system practices.

**Results:**

The study revealed a high awareness of the traffic light labeling system, with 89.8% of the participants recognising the labelling system. Over 80% of the participants correctly identified the colour codes (red, amber, and green) associated with high, medium, and low levels of sugar, salt, and fat. The participants expressed satisfaction with the clarity, adequacy, and helpfulness of the traffic-light system information. Ordinal regression analysis indicated that Sinhalese ethnicity, age ≥ 30 years, higher household income (> Rs. 50,000), and higher education levels were positively associated with adherence to the TLS best practices. Positive attitudes toward and good knowledge of the TLS significantly influenced purchasing behaviour.

**Conclusions:**

These findings suggest that the traffic light system is an effective tool for guiding healthier food choices among Sri Lankan consumers. However, targeted educational campaigns are needed to address knowledge gaps, particularly among older, lower-income, and less-educated populations. Continuous evaluation and refinement of the traffic light labeling system are recommended to maintain its efficacy and relevance in promoting public health.

**Supplementary Information:**

The online version contains supplementary material available at 10.1186/s12889-025-24994-1.

## Introduction

Noncommunicable Diseases (NCD) account for 63% of global mortality annually, with 75% of these deaths occurring in low- and middle-income countries (LMICs) [1]. In Sri Lanka, NCDs were responsible for 83% of deaths in 2018, highlighting the urgent need for interventions targeting modifiable risk factors such as unhealthy diets, physical inactivity, and tobacco use [2, 3]. To address this burden, Sri Lanka’s Ministry of Health aligned its National Multisectoral Action Plan for NCDs (2016–2020) with the World Health Organization (WHO) Global Action Plan, prioritising dietary reforms through reduced intake of salt, sugar, and saturated fats [2]. However, suboptimal diets driven by food environments that promote ultra-processed products remain a persistent challenge [4], necessitating evidence-based strategies to reshape consumer behaviours and industry practices [5].

The burden of diet-related NCDs, including cardiovascular diseases, diabetes, obesity, and certain cancers, is particularly high in South Asia [6], where shifts toward high-calorie, nutrient-poor processed foods have accelerated in recent decades [7]. Recent data suggest that dietary risks alone account for nearly eight million global deaths annually and are among the top three contributors to Disability-Adjusted Life Years (DALYs) [8]. These statistics emphasise the importance of targeted nutritional interventions in public health policy.

Nutrition labelling has emerged as a critical policy tool for combating diet-related, NCDs. While mandatory back-of-pack nutrition tables are widespread, the WHO advocates front-of-pack labelling (FOPL) as a more effective measure for guiding healthier choices [4]. Among FOPL systems, the traffic-light labelling system (TLS), which uses colour codes (red/amber/green) to signal “high”, “medium”, or “low” levels of key nutrients, has gained traction [9]. TLS not only simplifies decision-making but also incentivises product reformulation by industry actors [10]. Despite its potential, evidence of the real-world impact of the TLS remains mixed. Systematic approaches highlight their efficacy in improving health perceptions and directing consumer attention [11]; however, few studies have rigorously evaluated their influence on purchasing behaviour or long-term dietary shifts [9].

A meta-analysis by Ikonen et al. (2020) found that FOPL, including TLS, can significantly improve consumers’ ability to identify healthier options; however, the extent of behavioural change varies based on contextual factors such as label design, consumer demographics, and retail environments [12]. Similarly, a Canadian study involving 625 participants found that “High in” front-of-package labels significantly improved consumers’ ability to identify foods high in sugars, sodium, or saturated fat, and supported healthier food choices across varying levels of health literacy [13]. A comprehensive systematic review and network meta-analysis found that colour-coded labels, such as the TLS, significantly improved consumers’ selection of healthier foods and reduced the purchase of products high in sodium, fat, and saturated fat, supporting the effectiveness of mandatory FOPL policies [4].

Moreover, existing research disproportionately focuses on high-income countries, leaving critical gaps in understanding TLS implementation in LMICs [10]. Few studies in South Asia, including India and Nepal, have explored consumer understanding of and responses to nutrition labels. A 2023 study by Pokhrel in Nepal revealed that only 24% of youths consistently read nutrition labels on packaged foods, with factors such as perception, motivation, awareness, and health priorities influencing their behaviour [14]. Similarly, a study conducted in New Delhi found that while many consumers read nutrition labels on ultra-processed foods, the frequency of use remains irregular, with women being more likely to check labels than men. Although nutrition labels positively influence healthier food choices, common barriers such as limited time, lack of interest, and complex terminology reduce their effectiveness [15]. These findings emphasise the need for clearer, more accessible, and engaging nutrition labelling strategies to encourage healthier dietary habits in the region.

Sri Lanka’s 2016 TLS policy for sugar-sweetened beverages (SSBs), complemented by SSB taxation in 2017, provides a unique case study. Developed through collaboration among policymakers, nutrition experts, and civil society [16], the TLS framework was tailored to local dietary patterns and informed by global precedents, such as tobacco control [17]. By 2019, TLS was expanded to include salt and fat content, with plans to extend the labelling to restaurant menus and digital platforms [16]. Although these efforts reflect a progressive policy trajectory, challenges persist in rural outreach, industry compliance, and the measurement of long-term health outcomes [16].

Furthermore, few studies in South Asia have systematically explored the sociocultural and behavioural drivers of nutrition label use, including literacy levels, consumer trust in food systems, and affordability concerns, all of which may influence label interpretation and use [18]. Evidence from Sri Lanka and comparable LMIC settings is urgently needed to understand how such context-specific factors shape the success of interventions such as TLS.

Research gaps related to TLS remain twofold: limited evidence on TLS’s behavioural impact in real-world settings, particularly in LMICs, and scant data on how TLS interacts with socioeconomic and cultural factors to shape dietary choices [4, 9]. This study addresses these gaps by analysing TLS’s effects on consumer behaviour in Sri Lanka, contributing novel insights from the Global South to inform global NCD policy frameworks.

## Methods

We conducted a descriptive cross-sectional study in 25 districts in Sri Lanka from the 1 st of November 2022 to the 30th of April 2023. Food consumers in Sri Lanka were defined as individuals over 18 years of age who had purchased food. The sample size for this study was calculated using the formula provided by Lwanga and Lemeshow [19] with a 95% confidence interval and margin of error of 5%. Based on previous studies, we assumed an expected prevalence of awareness of the TLS of 50%, which was a conservative estimate to ensure a sufficient sample size. A design effect of two was applied to account for homogeneity within the clusters, which increased the final calculated sample size to 2,840 participants. Adjustments were made to account for potential non-responses, further ensuring the representativeness of the sample. Multistage cluster sampling and probability proportional to size (PPS) sampling techniques were used, combined with online recruitment, to collect data at a single point in time, consistent with a cross-sectional study design. Although the sampling approach involved multiple stages to ensure representativeness, the data collection provides a snapshot of participants’ characteristics and behaviors without any longitudinal follow-up. The overall response rate was 90.5% (*n* = 2,569), indicating robust participant engagement.

Sri Lanka is divided into 6918 public health midwife (PHM) areas. PHM is the smallest health administrative area in the country. A cluster was defined as a PHM area. A list of PHMs was obtained from the Ministry of Health, including their contact numbers. The number of PHM areas in each district was selected based on the PPS of the district’s population. The number of PHM areas (clusters) in each district was randomly selected for analysis. Thereafter, in the selected PHM area, five individuals > 18 years of age were randomly selected (random number method) by the area PHM. Both males and females were eligible for selection, ensuring gender inclusivity. The contact details of the selected individuals were obtained through registers maintained by the PHM, which usually provide comprehensive contact information for residents in their areas. The PHM subsequently contacted the individual via mobile phone, and a link for recruitment was sent to the selected individual’s phone.

A self-administered online questionnaire was used in Sinhala, English, and Tamil. The Google Form link was shared with each area’s PHM, who was responsible for selecting and contacting individuals from the official registers. The selected individuals were contacted via mobile phone, and a survey link was sent to their mobile phones. In instances where participants did not have direct internet access or smartphones, the PHM assisted by facilitating access through their own mobile devices or helped participants complete the Google form during home visits or community clinics. The purpose of this approach was to enhance inclusion and minimise selection bias. Section one of the questionnaire contained questions on sociodemographic and socioeconomic factors. Section two consisted of questions related to knowledge and Section three consisted of questions related to attitudes and practices.

Knowledge was assessed using three questions on the meaning of red, yellow, and green colors on food labels, with four response options for each as high, medium, low levels of nutrients, or “I don’t know.” One point was awarded for each correct answer, and zero points for incorrect responses. Participants scoring full marks (3/3) were classified as having “good knowledge,” while all others were categorized as having “less knowledge.” Attitude was measured through six items assessing perceptions of traffic light labels, rated on a five-point Likert scale ranging from “Not at all” to “Very much,” covering aspects such as helpfulness, clarity, and confusion. In addition, one overall question, “What is your attitude towards traffic light labeling?” was included, with response options from “Very important” to “Not important at all”, respondents selecting “Very important” or “Important” were considered to have a positive attitude. Practice was evaluated by asking participants how often they used traffic light labels when purchasing food, with five options from “Never” to “Always.” For analysis, responses were categorized into three groups as minimum use (Never/Rarely), moderate use (Sometimes), and best use (Most of the time/Always).

We used an ordinal regression analysis model to determine the factors influencing purchasing and TLS. The dependent variables were the minimum, moderate, and best practices. The best practices depicted the optimal practices (always and most of the time looking at the TLS while buying items) that influenced purchasing decisions. It should be noted that this operationalisation of “best practices” serves as a proxy measure of behavioural engagement with TLS, and does not directly capture the nutritional quality of food purchases or overall dietary intake.

**Fig. 1:**
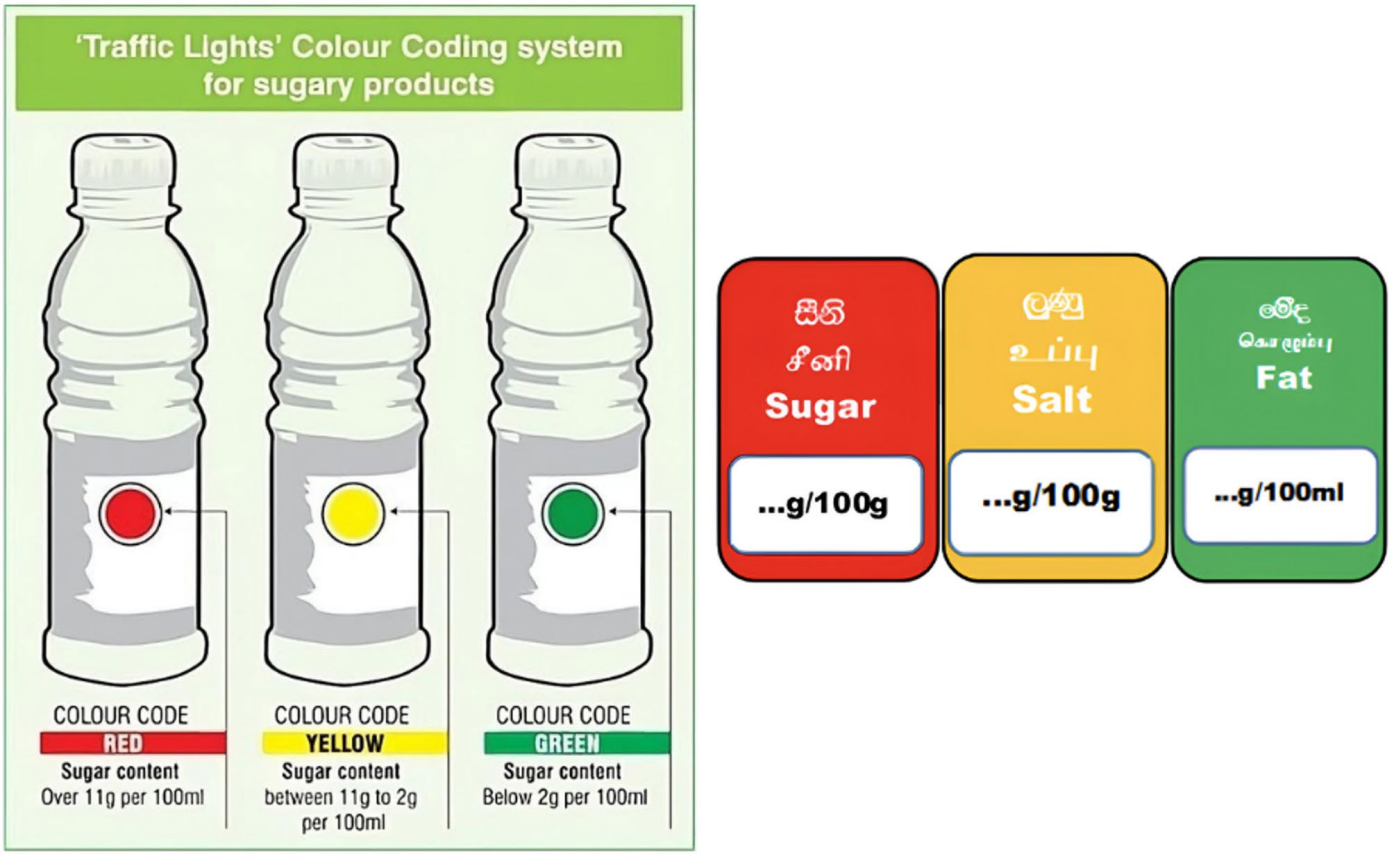
Front-of-pack labels used in Sri Lanka

The images shown in Fig. 1 were used as indicators of front-of-pack labels in Sri Lanka.

## Results

In this study, the response rate was 90.5% (*n* = 2569). The analysis of the sociodemographic data yielded several significant findings. In terms of age distribution, a considerable proportion of the respondents were relatively young, with 38.3% falling within the age range of 18-29 years. Males accounted for 20.2% and females for 79.8% of the respondents. The majority were Sinhalese (80.1%), followed by Tamil (14.6%). Regarding educational level, 73.1% of respondents had a satisfactory education level (A/L or above). When considering household income, the majority (68.8%) of households had an unsatisfactory monthly income. Interestingly, regarding the respondents’ level of awareness, 89.8% reported being aware of food products with a TLS (Table 1).

**Table 1:** Sociodemographic characteristics of the sample and level of awareness (*n* = 2569)

Variable	Number (%)
Age	
18–29 years	983 (38.3)
30–39 years	823 (32.0)
40–59 years	675 (26.3)
60 years or above	88 (3.4)
Sex	
Female	2051 (79.8)
Male	518 (20.2)
Ethnicity	
Sinhalese	2057 (80.1)
Tamil	375 (14.6)
Muslim	124 (4.8)
Burger	12 (0.5)
Other	1 (0.0)
Level of education	
Unsatisfactory education (Below A/L)	691 (26.9)
Satisfactory education (A/L and above)	1878 (73.1)
Monthly Household Income* (*n* = 2538)	
Unsatisfactory income (Rs.50000 or less)	1745 (68.8)
Satisfactory income (More than Rs.50000)	793 (31.2)
Ever seen the traffic light labeling system on any food item in Sri Lanka (*n* = 2569)	
Yes	2308 (89.8)
No	261 (10.2)
Ever heard of the traffic light labeling system in any food item in Sri Lanka (*n* = 261)	
Yes	63 (24.1)
No	198 (75.9)

The respondents’ level of knowledge clearly revealed that they had an excellent understanding of the colour codes used for TLS. An impressive 89.1% of participants correctly identified the red colour code, which denotes a high level of sugar, salt, or fat. Similarly, a significant number of participants (82.3%) correctly identified the green colour code, which denotes a low level of sugar, salt, and fat. Additionally, 83.9% of respondents correctly identified the amber (yellow) colour code, which represents a medium level of sugar, salt, and fat (Table 2).

Overall, the data indicated that a significant majority of the respondents had a strong understanding of the colour codes of the traffic-light labelling system. Specifically, 78.2% of the participants (*n* = 1851) demonstrated good knowledge of colour codes, suggesting that the system effectively communicates nutritional information to a large portion of the population. Conversely, 21.8% of the respondents (*n* = 517) had less knowledge of TLS, indicating the need for ongoing educational efforts to enhance understanding among this group.

**Table 2:** Knowledge based on the ability to correctly identify colors of the traffic light labeling system (*n* = 2368*)

	High level of sugar/salt/fat of the food product *n* (%)	Medium level of sugar/salt/fat of the food product *n* (%)	Low level of sugar/salt/fat of the food product *n* (%)	I do not have any idea *n* (%)
Red color code on food packaging	2111 (89.1%)	104 (4.4%)	40 (1.7%)	113 (4.8%)
Amber (Yellow) color code on food packaging	72 (3.0%)	1987 (83.9%)	165 (7.0%)	144 (6.1%)
Green color code on food packaging	56 (2.4%)	225 (9.5%)	1949 (82.3%)	138 (5.8%)

*Sample size varies between variables due to missing responses

The sociodemographic characteristics of the participants who reported not having good knowledge of TLS revealed several notable patterns. Among the 517 participants, a substantial majority (63.6%) were aged ≥ 30 years, indicating that a lack of knowledge was more prevalent in this age group [20]. In terms of household income, 83.9% earned Rs. 50,000 or less per month, suggesting that lower income may be associated with reduced knowledge of TLS. Ethnically, most of those with limited knowledge were Sinhalese (78.9%), with non-Sinhalese individuals constituting a small percentage (21.1%). The gender distribution revealed that 83.0% of the less knowledgeable participants were female and that 17.0% of the less knowledgeable participants were male. Interestingly, 56.9% of the participants with higher educational qualifications had less knowledge (Table 3).

**Table 3:** Sociodemographic characteristics of the participants without good knowledge of the traffic light labeling system (*n* = 517)

	Frequency	Percentage (%)
Age (*n* = 517)		
less than 30 years	188	36.4
30 years or more	329	63.6
Household Income (*n* = 509)		
Rs.50,000 or less	427	83.9
More than Rs.50,000	82	16.1
Ethnicity (*n* = 517)		
Non-Sinhalese	109	21.1
Sinhalese	408	78.9
Educational Level (*n* = 517)		
Below A/L	223	43.1
A/L and above	294	56.9
Gender (*n* = 517)		
Male Female	88 429	17.0 83.0

Several consumers mentioned that the information displayed on food packaging was adequate, with 37.3% mentioning that the information displayed on food packaging was “Quite a lot”, whereas 21.6% mentioned it as “Somewhat”, and 16.1% mentioned it as “very much.” When considering the confusion of the provided information, a substantial portion (31.7%) of participants found the information “Not at all” confusing, followed by 29.4% who found it “somewhat” confusing. Only a few participants (3.3%) said that it was “very much” confusing. Regarding the helpfulness of the information, the majority of consumers found the information on food packaging to be helpful, with 40.2% marking it as “quite a lot” and 30.5% as “very much”, while 3.5% marked it as “not at all”. A notable proportion of participants mentioned that the information on food packaging was both clear and understandable. For clarity, 39.9%, and for understandability, 39.6% were marked as “Quite a lot.” 45% of the participants mentioned that the information was “Not at all” misleading (Table 4).

**Table 4:** Level of information provided by the traffic light labeling system (*n* = 2371*)

	Not at all *n* (%)	A little bit *n* (%)	Somewhat *n* (%)	Quite a lot *n* (%)	Very much *n* (%)
Adequacy of information displayed	179(7.5%)	416(17.5%)	511(21.6%)	884(37.3%)	381(16.1%)
Whether the information provided is confusing	751(31.7%)	561(23.7%)	698(29.4%)	283(11.9%)	78(3.3%)
Helpfulness of information	82(3.5%)	206(8.7%)	406(17.1%)	954(40.2%)	723(30.5%)
Clarity of information	110(4.6%)	338(14.3%)	487(20.5%)	945(39.9%)	491(20.7%)
Understandability of information	90(3.8%)	334(14.1%)	426(18.0%)	939(39.6%)	582(24.5%)
Whether the information is misleading	1067(45.0%)	417(17.6%)	662(27.9%)	152(6.4%)	73(3.1%)

* missing = 198

Those who were Sinhalese (aOR = 2.52, 95% CI = 2.04, 3.11), aged ≥ 30 years (aOR = 1.68, 95% CI = 1.43, 1.98), had a household income ˃ Rs.50,000 (aOR = 1.21, 95% CI = 1.01, 1.45), and had an education level of A/L or above (aOR = 1.41, 95% CI = 1.16, 1.72) had greater odds of adhering to best practices when purchasing the product. Although gender was included in the model, males did not show a statistically significant difference in adherence compared to females (reference category) (aOR = 1.00, 95% CI = 0.82, 1.23, *p* = 0.976). Furthermore, individuals with positive overall attitudes toward TLS (aOR = 2.54, 95% CI = 1.95, 3.32) and good knowledge of TLS (aOR = 1.87, 95% CI = 1.54, 2.28) had greater odds of adhering to best practices while purchasing (Table 5). The ordinal regression model demonstrated excellent global fit, as evidenced by a highly significant likelihood ratio test [χ² = 277, *p* < 0.001], confirming the collective explanatory power of all predictors.

**Table 5:** Ordinal regression analysis of factors influencing traffic light labeling system (TLS) practices (order of the dependent variable; minimum, moderate and best practices)

	Adjusted Odds ratio	95% Confidence Interval		*p* value
		Lower Bound	Upper Bound	
Non-Sinhalese	Reference category			
Sinhalese	2.52	2.04	3.11	< 0.001
Female	Reference category			
Male	1.00	0.819	1.23	0.976
Age ˂30 years	Reference category			
Age ≥ 30 Years	1.68	1.43	1.98	< 0.001
Household income ≤ Rs.50,000	Reference category			
Household income ˃Rs.50,000	1.21	1.01	1.45	0.035
Education below A/L	Reference category			
Education A/L or above	1.41	1.16	1.72	< 0.001
Negative overall attitudes on TLS	Reference category			
Positive overall attitudes on TLS	2.54	1.95	3.32	< 0.001
Not having good knowledge on TLS	Reference category			
Having good knowledge on TLS	1.87	1.54	2.28	< 0.001

In a subsequent model that included theory-driven two-way interaction terms, we examined whether the effects of key predictors were modified by education, age, income, and attitudes, as suggested by prior studies. The following interaction terms were tested based on the literature: education × knowledge of TLS [21, 22], education × attitude toward TLS [23], income × attitude toward TLS [24], and age × knowledge of TLS [25]. Among these, only the interaction between household income and attitude toward TLS was statistically significant. Individuals with a monthly household income above Rs. 50,000 and positive attitudes toward TLS had significantly greater odds of adhering to the best purchasing practices (aOR = 3.39, 95% CI: 1.88,6.19, *p* < 0.001) (Supplementary Table). This finding suggests that the influence of positive attitudes is more likely to translate into action when economic constraints are less severe. The full results of the interaction model are available in the supplementary table.

## Discussion

This study offers critical insights into the behavioural and attitudinal impacts of the Sri Lankan TLS on food purchasing decisions, providing evidence on patterns associated with TLS use rather than causal relationships, addressing gaps in the evidence from low- and middle-income countries.

This study provides novel, large-scale evidence from Sri Lanka, a lower-middle-income country in South Asia, on consumer awareness, comprehension, and adherence to the TLS. Unlike previous studies predominantly conducted in high-income countries, our research captures the perspectives of diverse demographic groups in a LMIC context, including urban and rural populations, multiple ethnicities, and varied socioeconomic backgrounds. Notably, the study identifies key socioeconomic, demographic, and attitudinal determinants of TLS adherence, including interactions between income and positive attitudes toward TLS, offering insights into the conditions under which consumer engagement translates into behavioural practice.

These findings extend the global evidence base by highlighting that TLS effectiveness is moderated by contextual factors such as income, education, and cultural norms, which are highly relevant for other LMICs implementing front-of-package labelling policies [26]. Policymakers and public health practitioners can use this evidence to tailor nutrition labelling strategies and complementary interventions, including education campaigns, simplified messaging, and targeted outreach, to maximise effectiveness and equity in NCD prevention [27].

By providing context-specific evidence from the Global South, this study contributes to the broader discourse on front-of-package labelling as a tool for reducing diet-related non-communicable diseases worldwide.

### Demographic representativeness and implications

The high response rate (90.5%, *n* = 2569) highlights robust engagement, although the sample is skewed toward younger (38.3% aged 18–29), female (79.8%), and Sinhalese (80.1%) participants with higher education levels (40.1% holding diplomas or degrees) [28]. This contrasts with the general population of Sri Lanka (51% female, 14% aged 20–29 years) [28], suggesting a potential selection bias in this study. Despite this, the findings illuminate TLSs’ reach across urban and educated demographics while highlighting accessibility gaps in rural and lower-income groups.

The potential barriers to TLS usage in rural and low-income communities include limited nutritional literacy, economic constraints influencing food choices, reduced exposure to health promotion initiatives, and limited availability of TLS-labelled products in rural markets [29, 30]. Furthermore, cultural dietary practices and targeted food marketing strategies in low-income areas may contribute to deprioritising nutritional labelling [30]. These considerations offer a more comprehensive understanding of the factors underlying uneven awareness and adoption of TLS.

Moreover, this demographic skew mirrors findings in other LMICs and is supported by Ikonen et al. (2020), whose meta-analysis revealed that while FOPLs enhance consumers’ ability to identify healthier products, their influence on actual purchasing behaviour is more limited, highlighting that such effects are often shaped by underlying demographic and motivational factors such as education, income, and health awareness [12]. This pattern highlights the need for differentiated labelling and awareness strategies tailored to effectively reach underserved, rural, and lower-literacy populations. Public health campaigns using visual, symbol-based labelling, and community-based education interventions could potentially bridge this disparity.

However, the disproportionately higher representation of educated, urban, and female study respondents may reflect a population that is already predisposed to health literacy and behavioural responsiveness. This suggests that while TLS is effective among such groups, it may not achieve similar results among less literate or underserved populations without supportive interventions. Thus, the study’s findings should be interpreted in the context of this demographic skew, especially when generalising the behavioural impact of TLS across the entire population. In line with evidence from other LMIC studies, online or digitally assisted data collection methods often disproportionately capture younger, more educated, and urban participants, limiting the representativeness of the findings [31]. This pattern emphasise the importance of post-stratification weighting and mixed-methods recruitment in future research to ensure equitable inclusion of older adults, rural households, and lower socioeconomic groups [32]. Without such measures, studies risk overestimating awareness and responsiveness to nutrition labelling, as has been observed in South Asian and African settings [33].

Furthermore, it is important to emphasise that our definition of “best practices”, consistently looking at TLS while purchasing, reflects attentional engagement with nutrition labelling rather than a direct measure of healthier food purchases or improved diet quality. While engagement is a necessary precursor to informed decision-making, prior research indicates that label use does not always translate into actual healthier purchasing behaviour [34, 35]. Future studies should therefore validate this proxy indicator against objective purchasing data and dietary assessments to establish its potential predictive value for sustained health outcomes.

Consistent with this observation, our study also revealed a clear discrepancy between “awareness” and “adherence”, although awareness and comprehension of TLS were generally high, consistent adherence to healthy purchasing behaviours was uneven and associated with factors such as Sinhalese ethnicity, age ≥ 30 years, higher education, and higher household income. This gap was most evident among the 21.8% of respondents with “less knowledge” of TLS predominantly females, older adults, and lower-income participants indicating that knowledge alone does not guarantee behaviour change. This reinforces the need for targeted strategies addressing socioeconomic and cultural barriers to adoption, rather than relying solely on population-wide awareness campaigns [36].

### Awareness and comprehension of TLSs

Approximately 90% of the participants reported being familiar with TLSs, and over 80% accurately interpreted colour-coded nutrient thresholds. This aligns with evidence that TLSs simplify nutritional decision-making by reducing cognitive load, as demonstrated in previous eye-tracking studies [11]. Notably, the substitution of red-labelled items with healthier alternatives is correlated with reduced caloric, fat, and salt intakes, reinforcing the potential of TLSs for population-level dietary improvements [10].

These findings echo the global literature, which demonstrates improved consumer behaviour following the introduction of simple, interpretive labels such as the TLS and “High in” front-of-package labels [13]. The consistency of these outcomes across different settings strengthens the case for mandatory, colour-coded labelling systems in LMICs like Sri Lanka, where literacy and numeracy challenges persist. The capacity to accurately identify and understand the red, amber, and green codes indicates not merely superficial knowledge but a profound understanding of nutrient risk gradation. This suggests that visual labelling can address deficiencies in challenging nutritional literacy if the labels are contextually applicable and consistently applied. Furthermore, colour-coded nutrition labelling on traffic lights helps consumers with limited self-discipline to select healthier food options [37]. This suggests that standardising and simplifying the labelling content could minimise barriers linked to terminology complexity, a factor identified in other South Asian contexts [15].

The significance of this finding lies in its prospective applicability to other LMICs, where text-dense labelling may prove less useful owing to literacy constraints. Therefore, policymakers should consider integrating TLS with culturally adapted, simplified messaging, pictorial aids, and public awareness drives to maximise its public health impact.

### User satisfaction and usability

The participants expressed high satisfaction with TLS’s clarity and utility of the TLS: 37.3% rated information adequacy as “Quite a lot”, whereas 45% found labels “Not at all” misleading. These results mirror global findings, such as Hieke and Wilczynski’s report of high TLS understandability (5.9/7) [38] and Seward et al.’s observation that 59% of consumers found the TLS helpful [39]. However, Khalid’s caution against misleading labels underlines the need for standardised, culturally tailored TLS frameworks to ensure accuracy [40].

The TLS in Sri Lanka has demonstrated its potential as an effective behavioural nudge for consumers. A survey revealed that most Sri Lankan consumers have a satisfactory understanding of nutrition labels, including TLS [41]. Moreover, the finding that nearly one-third of consumers rated the sufficiency of labeling as considerable illustrates TLS’s practical applicability beyond mere theoretical understanding. This positive reception provides a platform for expanding TLS to other product categories currently exempt from labelling mandates, such as street foods and unpackaged items, which are widely consumed in LMIC settings.

The data indicate that TLS may affect both awareness and actual behavioural change, especially when accompanied by product reformulation and focused messaging. As suggested in recent systematic reviews (Ikonen et al., 2020; Jing Son, 2021), pairing FOPLs with complementary interventions, such as fiscal policies, media campaigns, and product placement strategies, could multiply their public health benefits.

### Determinants of TLS adherence

Ordinal regression identified Sinhalese ethnicity, age ≥ 30 years, household income ≥ Rs.50,000, and higher education level as predictors of TLS adherence. This finding suggests that socioeconomic and cultural factors mediate the effectiveness of labels. Among the 517 participants with limited TLS understanding, barriers included restricted access to nutrition education (especially in rural areas), reliance on unlabelled traditional diets, and cognitive challenges among older populations. Targeted interventions addressing these disparities could enhance equity in TLS adoption, as evidenced by studies linking nutritional literacy to purchasing behaviours [39, 42].

The comparatively lower adherence among non-Sinhalese populations, despite similar or increased health requirements, necessitates culturally sensitive interventions that respect linguistic diversity and regional dietary practices. This includes developing communication that appeals to ethnic minorities and customising educational materials to accommodate diverse cognitive and linguistic capabilities. Integrating multilingual labelling and health promotion materials could bridge this gap, as demonstrated by successful programs in multicultural LMIC contexts, such as India and Nepal [14].

Moreover, the absence of substantial gender disparity in TLS adherence, despite the majority of women in the sample, may indicate women’s established role in household food purchasing. This implies that enhancing women’s literacy regarding food labelling could yield significant improvements in household dietary behaviours [43]. Given women’s influence on household food choices, empowering them through targeted TLS education campaigns could serve as a strategic entry point for improving family level dietary patterns.

Additionally, interaction analysis revealed that individuals with higher income and positive attitudes toward TLS had significantly greater odds of adhering to best practices. This suggests that the influence of positive attitudes on label use may be amplified when economic constraints are relaxed, highlighting the combined importance of psychosocial readiness and financial capacity in enabling healthier purchasing behaviours.

Nevertheless, certain studies have indicated that consumer behaviour varies across different demographic groups and that there is an absence of a consistent correlation between nutritional quality and consumer behaviour [9, 44]. This emphasises the importance of integrating labelling initiatives with broader food environment reforms, such as regulating marketing, improving access to healthy options, and incentivising product reformulation to ensure sustained dietary improvements across all population segments.

### Strengths and limitations

The strengths of this study include its large sample size, multilingual data collection (Sinhala, English, and Tamil), and multistage cluster sampling to improve representativeness. The use of visual TLS simulations in questionnaires enhanced ecological validity by mimicking real-world shopping experiences. However, its cross-sectional design limits causal inference. Additionally, reliance on mobile-based recruitment and online data collection may have excluded individuals with limited digital access or low digital literacy, potentially introducing participation bias. Despite the assistance provided by PHMs, digital exclusion may have a disproportionate effect on individuals with a lower level of education or socioeconomic status. This digital exclusion likely resulted in underrepresentation of vulnerable groups, thereby potentially overestimating awareness and adherence to TLS in the general population. Furthermore, the possibility of self-selection bias, such as a greater likelihood of participation by health-conscious individuals, and recall bias may limit the generalisability of the findings to the broader population.

In addition, our operational definition of “best practices” (always or most of the time looking at the TLS while purchasing) measures behavioural engagement with the label rather than direct evidence of healthier purchasing decisions or improved dietary quality. While such engagement is a critical first step, future research should examine the extent to which adherence to TLS best practices translates into sustained changes in food purchasing, dietary patterns, and ultimately, reductions in NCD risk. Future longitudinal or experimental studies are warranted to confirm causal relationships between observed factors and TLS adherence and to better understand the directionality of these associations.

Although multistage cluster sampling and PPS techniques were employed, sample weighting was not applied. Therefore, the findings may not fully reflect the demographic distribution of the national population, which may limit the generalisability of the results. In particular, the sample was skewed toward younger, female, Sinhalese, and more highly educated respondents, who may already be predisposed to higher health literacy and behavioural responsiveness. As such, the results should be interpreted with caution and are most applicable to these demographic groups, rather than the wider Sri Lankan population. Future studies aiming for population-level inference should consider applying post-stratification weighting based on national census data.

## Conclusions and recommendations

This study demonstrates that the Sri Lankan TLS significantly shapes food purchasing behaviour, with high baseline awareness and understanding among consumers. The participants perceived the TLS as clear, adequate, and actionable, particularly in terms of urban and educational demographics. However, adherence to the TLS is influenced by socioeconomic and cultural factors. These findings emphasise TLS’s potential as a public health tool in LMICs, while highlighting disparities in access and understanding among rural, lower-income, and less-educated populations.

To maximise the impact of TLS in Sri Lanka, targeted interventions are required to address existing gaps and enhance equity. Promotional campaigns should be expanded to rural and marginalised communities, leveraging high urban awareness. These initiatives could include community-driven nutrition education programs delivered via local health officers, schools, and women’s groups, using simplified, visual TLS materials in Sinhala, Tamil, and local dialects to enhance comprehension among low-literacy and lower-income populations. Mobile-based outreach and community workshops can further improve reach to digitally excluded and older adults.

Policy makers should also incentivise food retailers in rural areas to stock TLS-labelled products and implement point-of-purchase nudges, such as shelf signage highlighting healthier options, to reinforce behavioural change. Targeted subsidies or pricing strategies for healthier products could reduce economic barriers to adherence among lower-income households.

Additionally, investing in longitudinal and equity-focused research is essential to understand the long-term impact of TLS. Periodic evaluations should monitor behavioural trends, dietary shifts, and NCD outcomes across different demographic groups to assess the effectiveness of interventions. Findings can inform culturally adapted and context-specific modifications to TLS implementation, ensuring equitable public health benefits.

## Supplementary Information

Supplementary Material 1.

## Data Availability

The datasets used in the current study are available from the corresponding author uponreasonable request.

## Abbreviations

NCD: Non-communicable diseases
TLS: Traffic light labeling system
LMIC: Low and middle-income countries
WHO: World Health Organization
FOPL: Front-of-Pack Labelling
SSB: Sugar-Sweetened Beverages
PPS: Probability Proportional to the Size
PHM: Public Health Midwife
DALY: Disability Adjusted Life Years
