# Supplementary material for: Understanding consumer response to front-of-package labeling: insights from a nationwide survey in Sri Lanka
Source: BMC Public Health. 2025 Oct 31;25:3687. doi: 10.1186/s12889-025-24994-1 (PMC12577392; doi:10.1186/s12889-025-24994-1)
Supplement: Supplementary file 1 — Supplementary Material 1. [file 12889_2025_24994_MOESM1_ESM.docx]

**Supplementary Table: Ordinal Regression Model including theory-driven interaction terms influencing adherence to TLS practices**

|  | **Adjusted Odds ratio** | **95% Confidence Interval** | | **p value** |
| --- | --- | --- | --- | --- |
|  |  | **Lower Bound** | **Upper Bound** |  |
| Non-Sinhalese | *Reference category* |  |  |  |
| Sinhalese | 2.57 | 2.08 | 3.17 | < 0.001 |
| Female | *Reference category* |  |  |  |
| Male | 0.99 | 0.81 | 1.21 | 0.979 |
| Age ˂30 years | *Reference category* |  |  |  |
| Age ≥30 Years | 1.63 | 1.44 | 2.31 | 0.007 |
| Household income ≤Rs.50000 | *Reference category* |  |  |  |
| Household income ˃Rs.50000 | 0.40 | 0.22 | 0.70 | 0.002 |
| Education below A/L | *Reference category* |  |  |  |
| Education A/L or  above | 1.89 | 1.04 | 3.45 | 0.037 |
| Negative overall attitudes on TLS | *Reference category* |  |  |  |
| Positive overall  attitudes on TLS | 2.06 | 1.28 | 3.37 | 0.003 |
| Not having good knowledge on TLS | *Reference category* |  |  |  |
| Having good knowledge on TLS | 2.01 | 1.30 | 3.10 | 0.001 |
| Education below A/L* Not having good knowledge on TLS | *Reference category* |  |  |  |
| Education A/L or  Above* Having good knowledge on TLS | 0.87 | 0.58 | 1.31 | 0.518 |
| Education below A/L* Negative overall attitudes on TLS | *Reference category* |  |  |  |
| Education A/L or  Above* Positive overall  attitudes on TLS | 0.79 | 0.44 | 1.43 | 0.450 |
| Household income ≤Rs.50000* Negative overall attitudes on TLS | *Reference category* |  |  |  |
| Household income ˃Rs.50000* Positive overall  attitudes on TLS | 3.39 | 1.88 | 6.19 | <0.001 |
| Age ˂30 years* Not having good knowledge on TLS | *Reference category* |  |  |  |
| Age ≥30 Years* Having good knowledge on TLS | 1.03 | 0.70 | 1.53 | 0.847 |
